# The First Case Report of a Patient With Oligodendroglioma Harboring *CHEK2* Germline Mutation

**DOI:** 10.3389/fgene.2022.718689

**Published:** 2022-02-23

**Authors:** Xueen Li, Hao Xue, Ningning Luo, Tiantian Han, Mengmeng Li, Deze Jia

**Affiliations:** ^1^ Department of Neurosurgery, Qilu Hospital of Shandong University and Brain Science Research Institute, Shandong University, Jinan, China; ^2^ The Medical Department, Jiangsu Simcere Diagnostics Co., Ltd, Nanjing Simcere Medical Laboratory Science Co., Ltd, The State Key Lab of Translational Medicine and Innovative Drug Development, Jiangsu Simcere Diagnostics Co., Ltd, Nanjing, China

**Keywords:** oligodendroglioma, germline mutation, *CHEK2* gene, potential genetic predisposition, CNS tumor

## Abstract

**Introduction:** *CHEK2* (Checkpoint kinase 2) germline mutations were associated with an elevated risk of breast cancer, colorectal cancer, and other familiar cancers. Loss-of-function variants in *CHEK2* are known to be pathogenic. Germline *CHEK2* mutations have also been observed in medulloblastoma and primary glioblastomas. Currently, there is no direct evidence supporting the relationship of *CHEK2* with central nervous system tumors.

**Case presentation:** A case of an oligodendroglioma patient harboring the germline *CHEK2* p.R137* mutation was reported. *CHEK2* p.R137* mutation occurred in the forkhead-associated domain. Given the absence of other known genetic predisposing risk factors, we considered that oligodendroglioma might be associated with the *CHEK2* mutation. The patient in our case might have a high risk of breast cancer and other multiple primary tumors. Her siblings and offspring would have a 50% chance of having the same variant.

**Conclusion:** We reported a case of an oligodendroglioma patient with a family history of gastrointestinal tumors harboring the germline *CHEK2* pathogenic variation. This is the first report of the association between the *CHEK2* pathogenic variation and brain tumors that warrants further validation in larger cohorts.

## Introduction

Checkpoint kinase 2 (*CHEK2*), a cell cycle checkpoint regulator gene and a tumor suppressor gene, is involved in DNA repair, cell cycle arrest, and apoptosis through the role in the homologous recombination DNA repair pathways (Stolarova et al., 2020). *CHEK2* is activated by an ataxia telangiectasia mutated (ATM) kinase and is in turn capable of phosphorylating several substrates including p53 and BRCA1, leading to cell cycle arrest, apoptosis, and DNA repair (Bartek and Lukas, 2003)*.* Heterozygous loss-of-function germline pathogenic variants in *CHEK2* are associated with an increased risk for breast cancer with a lifetime risk of 25–39% (Jalilvand et al., 2017).

*CHEK2* 1100delC allele is the most widely employed mutation point and is associated with Li-Fraumeni syndrome. Missense mutation I157T, another common *CHEK2* pathogenic variant, is reported to be associated with a higher risk for colon cancer (Jalilvand et al., 2017). The effects of *CHEK2* truncating and missense mutations might be different. The association between *CHEK2* and other cancers, including the male breast, kidney, gastric, prostate, lung, ovarian, and thyroid cancers, was also reported (Cybulski et al., 2004). Germline *CHEK2* mutations have also been observed in medulloblastoma and primary glioblastomas (Sallinen et al., 2005). Currently, there is no direct evidence supporting the relationship of *CHEK2* with CNS tumors. Herein, we reported a p.R137* mutation that occurred in the *CHEK2* FHA domain in a Chinese female patient with oligodendroglioma for the first time.

## Case Presentation

A 40-year-old female was admitted to hospital on 30 June 2020 for sudden loss of consciousness for 17 days. Physical examination showed binocular vision decline. Brain magnetic resonance imaging (MRI) was performed, and it showed irregular and abnormal signal shadows in the right frontal lobe, low signal in T1 and high signal in T2, no obvious enhancement in lesions on enhanced scan, mild compression of the surrounding brain tissue, and no significant shift in the midline structure. Diagnosis on admission showed the right frontal lobe space occupying the lesion and possibly symptomatic epilepsy. After the exclusion of surgical contraindications, the right frontal lobe glioma was removed under general anesthesia on 3 July 2020.

**FIGURE 1:**
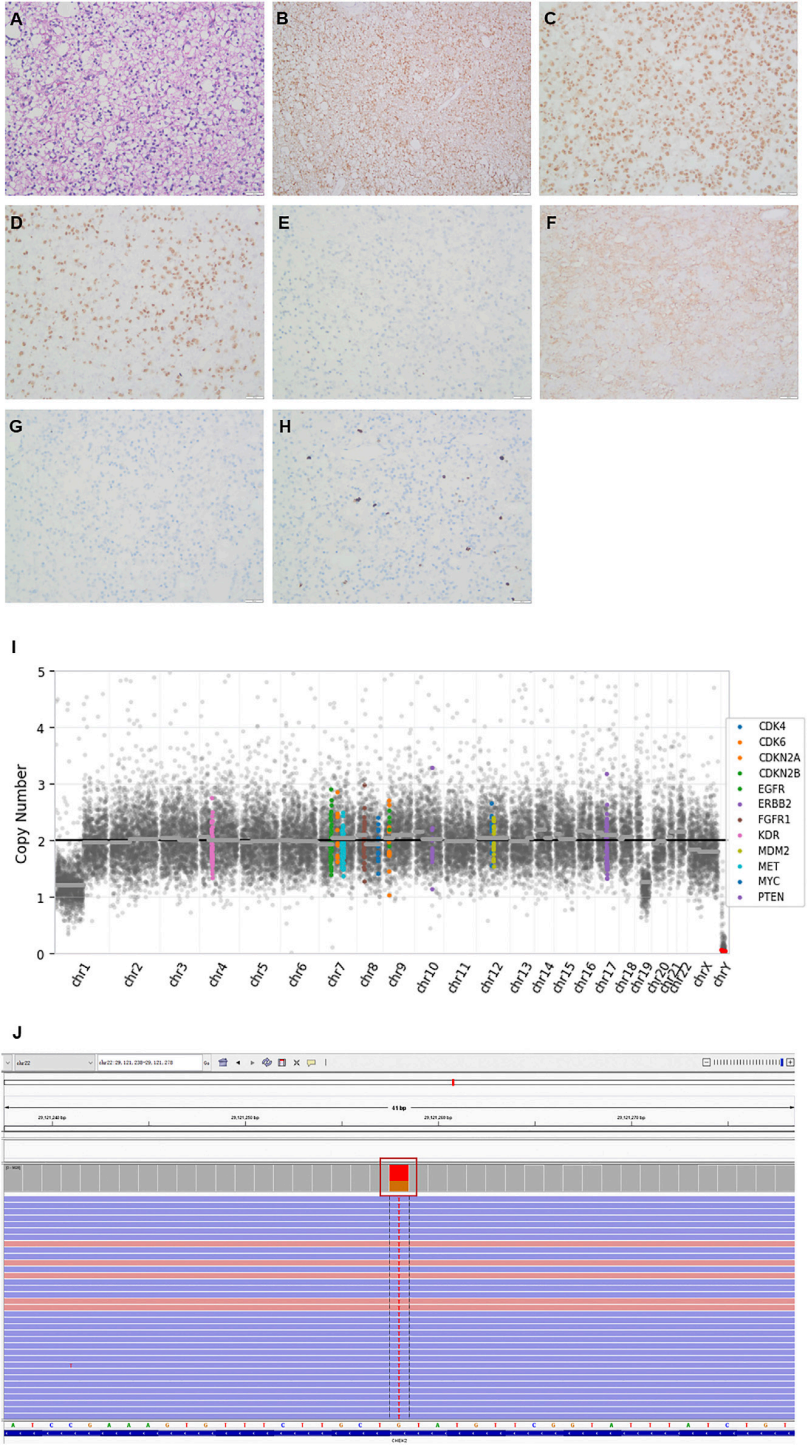
Histopathologic stains from the postoperative right frontal lobe **(A**–**H)**, 1P19Q chromosomal combination deletion **(I)** and the Integrative Genomics Viewer snapshot of CHEK2 p.R137* **(J)**. **(A)** hematoxylin and eosin. **(B)** IDH. **(C)** ATRX. (D) Olig2. **(E)** P53. **(F)** GFAP. **(G)** NeuN. **(H)** Ki67. (200×).

Postoperative pathology showed that the morphology of the right frontal lobe was consistent with oligodendroglioma (WHO Grade II), IDH (+), ATRX (+), Olig-2(+), P53 (−), GFAP (−), NeuN (−), and ki-67 (10%) (Figure 1). Next-generation sequencing (NGS) 131+4 panel (Simceredx) profiling was performed using the postoperative tissue, and *IDH1* R132H (allele frequency, AF 35.1%), *TERT* C228T (AF 38.02%), and 1P19Q chromosomal combination deletions (Figure 1I) were identified, which indicated that the tumor had typical molecular characteristics of oligodendroglioma. Moreover, *CHEK2* p.R137* (AF 49.36%) was also detected in the tumor tissue. Then, considering the family history of tumors, we confirmed the mutation as a germline heterozygous variation using leukocytes (Figure 1J). Somatic mutation of *CHEK2* and the deleterious variant of the *TP53* gene were absent.

**FIGURE 2:**
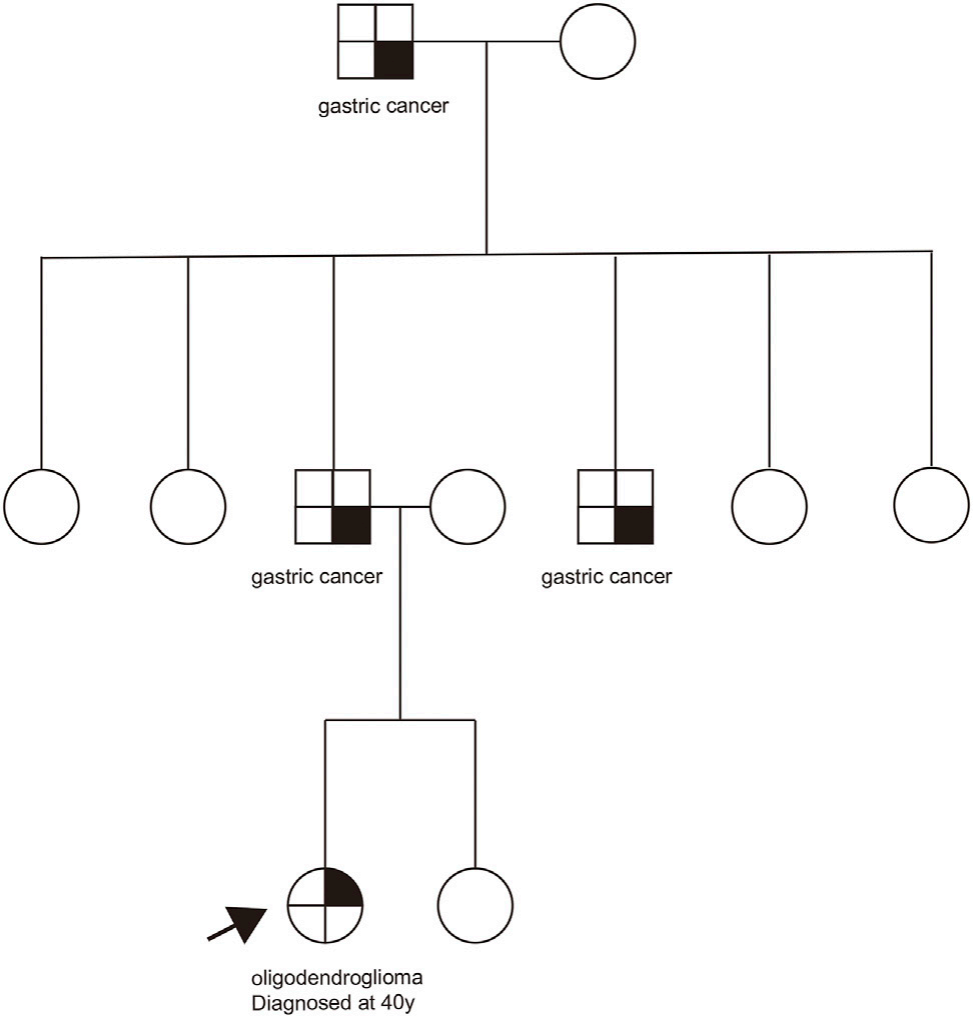
Pedigree chart of this family with cancer history.

The patient had no history of other tumors, and her grandfather, father, and uncle all suffered from gastric cancer in her family (Figure 2). The family history of the patient excluded Li-Fraumeni/Li-Fraumeni–like syndrome (Giacomazzi et al., 2015). Due to the death of the tumor patients in the family, further family co-segregation analysis was not conducted.

One month after surgery, considering that the patient was older than 40 years old and in accordance with the patient’s wishes, three-dimensional conformal radiotherapy (54 Gy/27F) and concurrent temozolomide chemotherapy (120 mg/qd) were administered. One month after concurrent chemoradiotherapy, six cycles of 240 mg/qd adjuvant temozolomide chemotherapy were given on days 1–5 every 4 weeks. At the time of writing, the patient has been discharged from the hospital and returned to work. According to the National Comprehensive Cancer Network (NCCN) guidelines, a woman with a germline *CHEK2* pathogenic variant has an estimated 15–40% overall risk of breast cancer, and an annual mammogram and consideration of tomosynthesis and breast MRI with contrast were recommended for the patient in our case.

## Discussion

*CHEK2*, a serine/threonine kinase gene, plays an essential role in the DNA damage checkpoint pathway, which can prevent damaged cells from entering mitosis or arrest the cell cycle (Apostolou and Papasotiriou, 2017). *CHEK2* is also required for chromosomal stability during mitosis (Stolz et al., 2010). The p.R137* mutation occurred in the functionally important domain of C*HEK2* (the FHA domain), resulting in the loss of the protein kinase domain and promoting tumor formation. *CHEK2* p.R137* has been previously seen in very low population frequencies in TOPMed, gnomAD, and ExAC. This mutation is listed in the ClinVar database (https://www.ncbi.nlm.nih.gov/clinvar/variation/182452/) and has only been observed in individuals affected with breast cancer (Leedom et al., 2016), and this case would be an informative addition.

Although it is not clear at present if the *CHEK2* p.R137* variant is associated with the oligodendroglioma development in this patient, the heterozygous germline *CHEK2* or *TP53* pathogenic variants have been reported in patients with brain tumors (Bougeard et al., 2015; Valdez et al., 2017; Aedma and Kasi, 2020). In consideration of the unavailability of family patient samples, the relationship between *CHEK2* mutations and CNS tumors needs more research and reports in the future. Our case is a patient with an oligodendroglioma phenotype harboring the *CHEK2* p. R137* mutation, suggesting her siblings and offspring would have a 50% chance of having the same variant, which may increase the tumor risk.

The treatment options for oligodendroglioma are limited. Usually, primary surgery followed by postoperative radiotherapy and chemotherapy is performed (Weller et al., 2017). *CHEK2* is an important gene in the DNA homologous recombination repair (HRR) pathway. Inactivation of the ATM-CHEK2 pathway has been reported to sensitize human tumors to radio/chemotherapy based on cisplatin, temozolomide, and poly (ADP-ribose) polymerase inhibitors (Manic et al., 2015). Currently, the FDA has approved olaparib for metastatic castration-resistant prostate cancer patients with defective HRR genes. In the future, olaparib combined with temozolomide/radiotherapy might be a promising treatment option for patients with CNS tumors harboring the *CHEK2* germline mutation.

In our case, there are some limitations. This family has no cancers typically associated with the *CHEK2* pathogenic variant, and in this family, the patient’s grandfather, father, and uncle all expired, so the pedigree verification was not conducted. There was no loss of heterozygosity (LOH) found in the tumor tissue in our case. Therefore, further studies are needed to confirm the association between the *CHEK2* gene and oligodendroglioma.

## Conclusion

To our knowledge, this is the first report that we identified a pathogenic variant *CHEK2* p.R137* in an oligodendroglioma patient, which suggests that brain tumor patients with a family history of cancer or other risk factors need to pay more attention to the potential genetic predisposition variant for moderate/low penetrance genes. This may provide the direction for future treatments.

## Data Availability

The original contributions presented in the study are included in the article/Supplementary Materials, further inquiries can be directed to the corresponding author.
